# Novel serum proteomic signatures in a non-human primate model of retinal injury

**Published:** 2011-03-23

**Authors:** Jeffrey J. Dunmire, Rachida Bouhenni, Michael L. Hart, Bassam T. Wakim, Anthony M. Chomyk, Sarah E. Scott, Hiroshi Nakamura, Deepak P. Edward

**Affiliations:** 1 Department of Ophthalmology, Summa Health System, Akron, OH; 2 Department of Biochemistry, Medical College of Wisconsin, Milwaukee, WI

## Abstract

**Purpose:**

To identify candidate protein biomarkers in sera indicative of acute retinal injury.

**Methods:**

We used laser photocoagulation as a model of acute retinal injury in Rhesus macaques. In a paired-control study design, we collected serum from each animal (n=6) at 4 h, 1 day, and 3 days following a mock procedure and then again following retinal laser treatment that produced mild lesions. Samples were fractionated by isoelectric focusing, digested with trypsin, and analyzed by liquid chromatography/tandem mass spectrometry (LC-MS/MS). Spectral counting was used to determine relative protein abundances and identify proteins with statistically significant differences between control and treated sera.

**Results:**

Mild retinal injury was confirmed by fundus photography and histological examination. The average number of total proteins detected by LC-MS/MS was 908±82 among samples from all three time points. Following statistical analysis and employing stringent filtering criteria, a total of 19 proteins were identified as being significantly more abundant in sera following laser-induced retinal injury, relative to control sera. Many of the proteins detected were unique to one time point. However, four proteins (phosphoglycerate kinase 1, keratin 18, Lewis alpha-3-fucosyltransferase, and ephrin receptor A2) showed differences that were significant at both 4 h and 1 day after laser treatment, followed by a decrease to baseline levels by day 3.

**Conclusions:**

A serum biomarker response to mild retinal laser injury was demonstrated in a primate model. Among the proteins detected with highest significant differences, most are upregulated within 24 h, and their appearance in the serum is transient. It is conceivable that a panel of these proteins could provide a means for detecting the acute-phase response to retinal injury. Further investigation of these candidate biomarkers and their correlation to retinal damage is warranted.

## Introduction

Retinal proteins have been found in the serum of patients with conditions such as retinal detachment and diabetic retinopathy [1,2]. However, the identification of acute-phase biomarkers following retinal injury has not been described. We hypothesized that retinal injury by laser photocoagulation causes death and/or injury of photoreceptor and retinal pigment epithelium (RPE) cells, and that proteins that may be upregulated in response to the damage will be released from those, or adjacent, cells into the systemic circulation. Detection of such proteins in serum would allow the identification of candidate biomarkers. A panel of serum biomarkers could provide the basis for the development of a rapid and minimally invasive diagnostic test to detect acute retinal injury.

Laser-induced retinal damage for energy levels above the clinically detectable threshold has been well described. Retinal damage can be localized to deep or superficial layers, or might involve the entire retinal thickness, and has been extensively studied in animal models [3-5]. The mechanism of damage may be photothermal, photomechanical, photodisruptive, or photochemical [3]. Irrespective of the mechanism of damage, laser-tissue reaction leads to varying degrees of retinal neuronal and RPE cell damage or death [3-7]. It also leads to altered protein expression in the retina and disruption of the outer blood-retinal barrier [8-12]. It is conceivable that such events would lead to the leakage of proteins from the damaged photoreceptor/RPE cells and/or surrounding tissue through the underlying choroid and into the systemic circulation.

We sought to discover whether retinal injury could cause detectable leakage of proteins from the retina into the systemic circulation. Using a proteomics approach, we investigated whether detection of this acute-phase response process was possible over a three day course of time following injury. Liquid chromatography-tandem mass spectrometry (LC-MS/MS) analysis of complex biologic fluids is a highly sensitive method for observing qualitative and quantitative changes in protein content. Others have used LC-MS/MS-based proteomics as a means of detecting changes due to ocular disease [2,13] and the technique is used extensively for biomarker discovery. We used laser photocoagulation as a model of acute retinal damage in non-human primates followed by LC-MS/MS analysis to identify candidate protein biomarkers of retinal injury in serum.

## Methods

### Animals

Seven female Rhesus macaques (*Macaca mulatta*), aged 2.5–3.5 years and weighing 3.0–4.5 kg, were used in this study. One animal was used for clinicopathologic correlation of laser injury and the remaining six were treated in a paired-control fashion. This study was conducted in accordance with the Association for Research in Vision and Ophthalmology’s Statement for the Use of Animals in Ophthalmic and Vision Research, and all experimental procedures were approved by the Institutional Animal Care and Use Committee at Northeastern Ohio Universities Colleges of Medicine and Pharmacy.

All animal procedures were performed under general anesthesia using 10 mg/kg ketamine and 0.075 mg/kg medetomidine, delivered intramuscularly. Upon completion of the procedures, 0.075 mg/kg atipamezole was administered as a reversing agent.

### Laser treatment

We studied the serum biomarker response from retinal laser lesions ranging from mild to minimally visible lesions (MVLs). These retinal laser lesions appeared light gray to yellow in color immediately after the injury. It has been reported that these MVLs are not visible one week after laser exposure [3,5,7,14]. In one animal, we tested the ability to create MVLs that could be confirmed clinically and upon pathologic examination. Higher intensity laser burns were created as rows parallel to MVLs to aid in their identification upon clinical and pathologic examination. Fundus images were taken immediately following laser treatment. The eye was enucleated 24 h later, following euthanasia, for histological examination. The whole globe was fixed in 10% neutral buffered formalin, after which the retinal area containing lesions was carefully dissected and oriented to ensure both lesion types could be observed in the sections. The tissue was processed, embedded in paraffin, sectioned, and stained with hematoxylin and eosin. The experimental mock control and MVL procedures were then performed, as described below.

Each animal in this experiment served as its own control by undergoing a pre-treatment mock procedure. In this way, a baseline serum proteome was established for each animal, allowing any changes that may have occurred due to anesthesia, pupil dilation, or handling to be excluded. Following general anesthesia, the pupils were dilated with one drop each of tropicamide 1.0% (Bausch & Lomb, Tampa, FL) and phenylephrine HCl 2.5% (Alcon Laboratories, Fort Worth, TX), and one drop of proparacaine HCl 0.5% (Bausch & Lomb) was instilled as a local anesthetic. Eyes were examined by indirect ophthalmoscopy to rule out the presence of any retinal abnormality or other ocular disease. Three weeks following the control experiment, the animals were treated in a similar fashion, except that the right eye of each animal received laser treatment. Laser photocoagulation was performed with a frequency-doubled Nd: YAG 532 nm laser (OcuLight^®^GL; IRIDEX Corporation, Mountain View, CA). A total of 15 laser spots were applied to the retina superior to the optic nerve in the right eye. The laser settings were 500 µm spot diameter, 130–160 mW power intensity, and 0.1 s duration. These parameters consistently produced MVLs, as described previously.

### Sample collection

Blood was collected by venipuncture, under general anesthesia, at 4 h, 1 day, and 3 days following both the mock procedure and the laser treatment. At each time point, approximately 5 ml of blood was drawn into collection tubes free of clot activator or other additives. After clotting at room temperature, the blood was centrifuged at 2,500× g for 10 min for serum separation. The collected serum was aliquoted to 1.5 ml tubes and stored at −80 °C until used for proteomic analysis.

### Sample preparation and mass spectrometry

Knowing that protein concentrations in serum span a dynamic range of greater than 10 orders of magnitude [15,16] and that the 6–10 highest abundance serum proteins, which include albumin and globulin, account for greater than 90% of the total protein content [17,18], it is imperative to employ sample pre-fractionation before conducting mass spectrometry.

Albumin and globulin depletion is one way to reduce the complexity of serum, but may also lead to the depletion of potentially important protein targets and non-specific loss of low abundance proteins [19,20]. We elected to reduce the complexity of the serum by subjecting it to isoelectric focusing (IEF), which does not involve the removal of any proteins. This process, when compared to that without fractionation, led to the identification of six times the number of proteins using mass spectrometry. The use of IEF increased both the protein detection and identification rates to a degree that helped determine differences between the serum proteomes of control and treated animals.

The serum samples were pre-fractionated offline by liquid-phase IEF using a MicroRotofor™ (Bio-Rad Laboratories, Hercules, CA). Fifty microliters of each sample were focused in the range between pH 3–10, producing ten fractions that were each collected in a final volume of 200 µl. One hundred microliters from each serum fraction was processed as described by Yu et al. [21], with minor changes to the wash steps, as follows. In brief, gel pieces were incubated with 40% methanol + 7% acetic acid for 30 min, washed four times in water for 30 min under sonication, washed once in 200 mM ammonium bicarbonate (pH 8) and twice in 50% acetonitrile, 100 mM ammonium bicarbonate (pH 8). Following drying of the gel pieces, each was incubated overnight at 37 °C in a solution of 100 mM ammonium bicarbonate (pH 8) containing 0.5 µg trypsin (Promega Corporation, Madison, WI). Peptides were extracted twice from the gel pieces using 70% acetonitrile and 0.1% formic acid and then dried. Extracts were resuspended in 20 µl of 6 M guanidine-HCl, 5 mM potassium phosphate, and 1 mM DTT (pH 6.5), sonicated, and passed through a C18 ZipTip (Millipore, Billerica, MA) to extract the peptides, which were then dried. Prior to mass spectrometry, the dried peptide extracts were dissolved in 5 µl of 5% acetonitrile and 0.1% formic acid.

Peptides from each IEF fraction were analyzed once by automated nano-flow liquid chromatography tandem mass spectrometry (nano-flow LC-MS/MS) using an LTQ linear ion trap mass spectrometer (Thermo Fisher Scientific, Waltham, MA), coupled to a Surveyor HPLC system (Thermo Fisher Scientific) equipped with a Finnigan Micro AS autosampler, and interfaced with an Aquasil C18 PicoFrit 75 µm×10 cm capillary column (New Objective, Woburn, MA). Peptide mixtures were first separated using the C18 reverse phase column (1 µl/min flow rate) in line with the mass spectrometer. Mobile phases consisted of 0.1% formic acid, 5% acetonitrile and 0.1% formic acid with 95% acetonitrile for solvents A and B, respectively. A linear gradient of 180 min was followed by 60 min equilibration in solvent A. Ions eluted from the column were electrosprayed at a voltage of 1.75 kV. The LC-MS/MS cycle was 6 MS/MS scans per full MS scan. Dynamic exclusion enabled ±1.5 Da tolerance and 12 s exclusion duration.

### Data analysis

Data generated from the raw spectra was searched against the Macaca mulatta subset of the UniProt database using the Sequest (V.12) program using a peptide mass tolerance of 2.5 Da, a fragment mass tolerance of “0” (which is effectively 1 Da), and monoisotopic masses.

Probability scores were calculated using Epitomize software [22,23] (Medical College of Wisconsin, Milwaukee, WI) and filtered at 0.85 and 0.60 for spectra and protein hits, respectively. Additionally, proteins identified by less than two unique peptides were eliminated and not considered for further statistical analysis. The protein hits from each set of ten IEF fractions for each sample were combined to represent the serum proteome of that specimen at the given time point. For each time point, the data results from all control and treated specimens were quantitatively compared using Visualize software [22,23] (Medical College of Wisconsin, Milwaukee, WI). A normalized p value for each protein was calculated using the G test as previously described [24]. These results were compiled into full and un-edited lists of proteins, before application of any further statistical criteria or filtering, and are available as supplemental data in Appendix 1, as described below in the Results section. Proteins with a normalized p value <0.05 were filtered to identify those detected either in treated samples only or with increased abundance in treated samples relative to the controls. The p values for this subset of data were adjusted according to the Holm-Sidak method of correction for multiple comparisons. Proteins with an adjusted p value <0.05 were considered significant and were retained. Proteins were eliminated from the list if the normalized scan count ratio did not represent at least a twofold increase in the treated group relative to the control. Additionally, proteins were filtered based on the frequency of detection; they were not included in the final list, despite their statistical significance, unless detected in at least 50% of the treated samples. The stringent statistical criteria and sorting methods were chosen to increase confidence in the final panel of candidate protein biomarkers. These choices helped to eliminate proteins that may have been specific only to individual animals or that may have been unrelated to the injury. Using our method, we minimized the rate of false identification and compiled a list of proteins that were both significantly present in each sample and significantly different between treatment groups. These methods for the comparative analysis of proteomic data sets have been successfully used in our laboratory, as described in a recent publication [25].

Protein lists for each time point were analyzed using Pathway Studio software (Ariadne, Rockville, MD). Using the human homolog gene identifiers, each protein was mapped to its associated gene ontology (GO) terms for both cellular components and biologic processes. The GO terms for each category were analyzed for enrichment. Terms common to the greatest number of proteins were used as the defining terms in each category.

## Results

### Histopathological assessment of laser lesions

**Figure 1:**
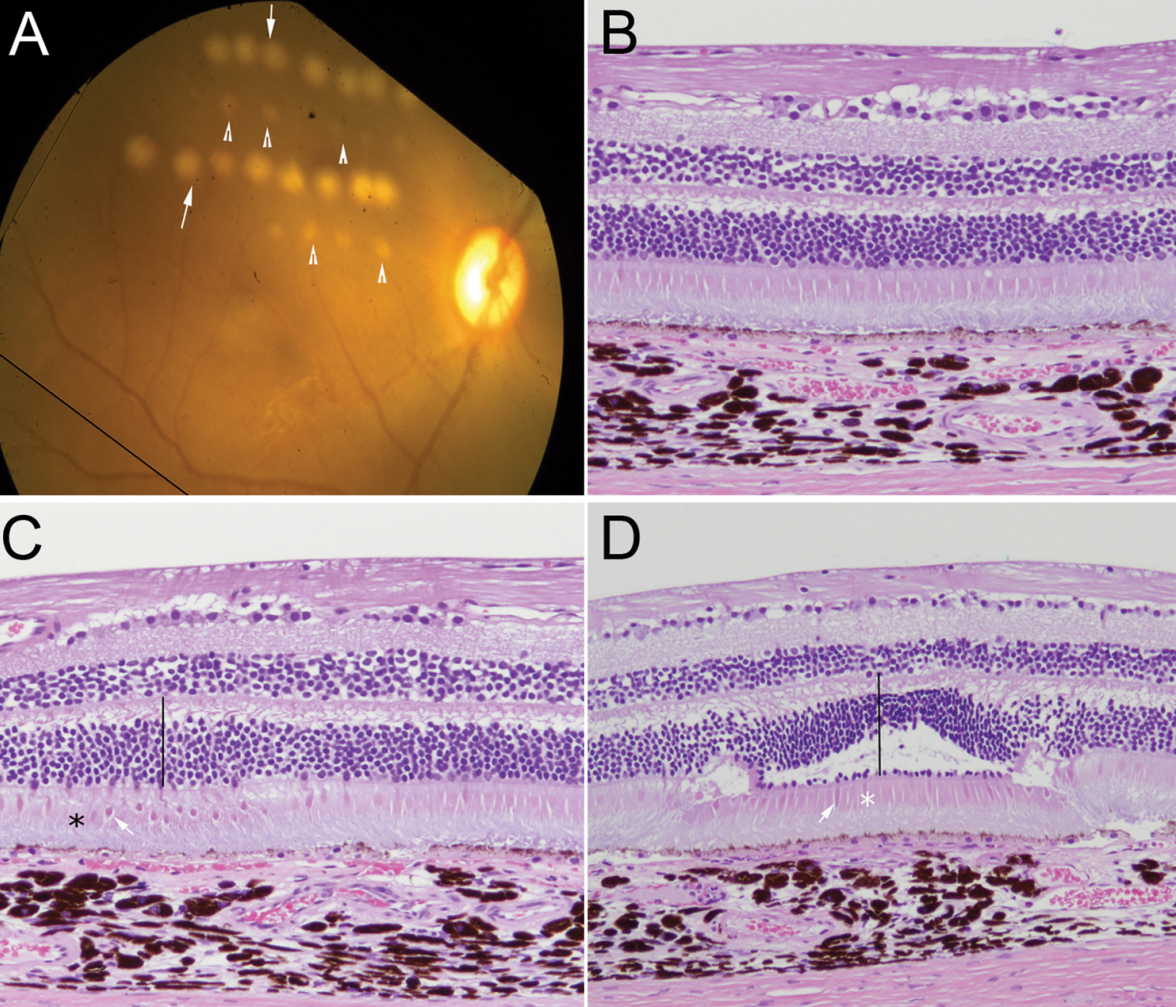
Fundus examination and histological evaluation of severe and MVL type laser lesions in a retina. **A**: Rows of severe lesions (arrows) and MVL (arrow heads) caught by fundus imaging immediately following laser treatment. **B**-**D**: Histology of normal and laser-treated retina 1 day following laser injury. **B**: Normal retina adjacent to the region that was treated with laser. Note the intact sensory retina and retinal pigment epithelium. **C**: Minimally visible lesions show mild swelling of the outer nuclear layer (line), condensation of cone inner segments (arrow), and mild disruption of photoreceptor outer segments (*) and RPE. The inner retina and underlying choroid are unaffected. **D**: A severe retinal lesion showing outer retinal swelling with disruption of the outer nuclear layer, the outer plexiform layer, and portions of the inner nuclear layer (line). Note the mummification of photoreceptor inner segments (*), as indicated by shrinkage and condensation (arrow). Also note mummification [69,70] of the underlying photoreceptor outer segments and RPE (hematoxylin and eosin, original magnification 20×).

We confirmed the ability to create MVLs and mild type laser lesions, as demonstrated by fundus photography and histological examination. The pathologic changes associated with MVLs or severe type lesions were distinct and clearly identifiable in histological sections of treated retinas at 1 day after laser injury and when compared to normal retinas (Figure 1).

### Protein identification and normalized scan count ratio

The total number of proteins detected by LC-MS/MS analysis were 818, 928, and 978 at the 4 h, 1 day, and 3 days time points, respectively. A comprehensive list of these proteins, identified by two or more non-redundant peptides, appears in Appendix 1 as supplemental data. These protein identifications served as the starting point for determining a final list of candidate biomarkers. For biomarkers of retinal injury, we chose to consider only those proteins that were either present in treated samples only or that showed increased abundance in treated samples relative to controls. In this case, the numbers of proteins considered for further statistical analysis and data filtering were 259, 189, and 353 for the 4 h, 1 day, and 3 days time points, respectively. The numbers of proteins that passed the additional statistical testing and met the final filtering criteria were 8, 12, and 3 for the 4 h, 1 day, and 3 days time points, respectively (Table 1). Based on their normalized scan count ratios, these proteins were identified as being considerably more abundant in sera following laser treatment.

**Table 1:** Proteins with significantly higher spectral counts in serum following laser treatment compared with control.

Time post-treatment	*Macaca mulatta* Uniprot ID	Human homolog gene ID	Protein description	Number samples w/ positive detection		Normalized scan count ratio (treated/control)	Holm-Sidak adjusted p-value
				Control (n=6)	Treated (n=6)		
4h	Q8SPT9	3875	Keratin 18 (CK18)	1	6	16.8935	0.000783
4h	Q3YAQ9	5230	Phosphoglycerate kinase 1 (PGK1)	5	6	5.0837	0.000309
4h	Q6XML5	721	Complement factor 4 (C4)	6	6	2.7795	0.019637
4h	Q8WNP0	2525	Lewis alpha-3-fucosyltransferase (FUT3)	6	6	2.2793	0.002356
4h	Q1HKZ4	1969	Ephrin receptor A2 (EPHA2)	6	6	2.2169	0.003633
4h	Q9TUC6	1138	Nicotinic receptor alpha 5 subunit (CHRNA5)	0	5	-	0.003949
4h	Q28864	7035	Tissue factor pathway inhibitor (TFPI)	0	4	-	0.029670
4h	B0JDR3	3106	MHC class I antigen (HLA-B)	0	3	-	0.001778
1 Day	Q3YAQ9	5230	Phosphoglycerate kinase 1 (PGK1)	2	6	24.6994	2.62E-13
1 Day	Q8SPT9	3875	Keratin 18 (CK18)	2	6	22.7121	5.42E-12
1 Day	Q8WNP0	2525	Lewis alpha-3-fucosyltransferase (FUT3)	5	6	15.3307	0.00E+00
1 Day	B1NL87	64816	Cytochrome P450, 3A43 (CYP3A43)	3	6	6.2458	0.013228
1 Day	A9XEK3	146	Alpha-1D adrenoceptor (ADRA1D)	4	5	6.0684	0.00E+00
1 Day	P47899	153	Beta-1 adrenergic receptor (ADRB1)	3	4	4.3532	0.000097
1 Day	Q5TM61	5514	Protein phosphatase 1, regulatory subunit 10 (PPP1R10)	5	6	4.3240	4.47E-07
1 Day	B3Y660	51311	Toll-like receptor 8 (TLR8)	6	6	3.2290	0.00E+00
1 Day	Q1HKZ4	1969	Ephrin receptor A2 (EPHA2)	6	6	2.7874	0.000157
1 Day	Q8HYQ1	6352	C-C motif chemokine 5 (CCL5)	0	6	-	0.000238
1 Day	Q3YAN2	51185	Cereblon (CRBN)	0	6	-	0.016533
1 Day	Q4G3V3	793	28kDa calbindin 1 (CALB1)	0	6	-	0.041927
3 Days	Q50KV9	54429	Taste receptor type 2 (TAS2R5)	3	5	5.6981	0.002844
3 Days	B0S4P2	148	Alpha-1A adrenoceptor (ADRA1A)	4	6	5.2097	0.011880
3 Days	Q9N143	7297	Tyrosine kinase-2 (TYK2)	6	6	2.2057	0.021489

**Figure 2:**
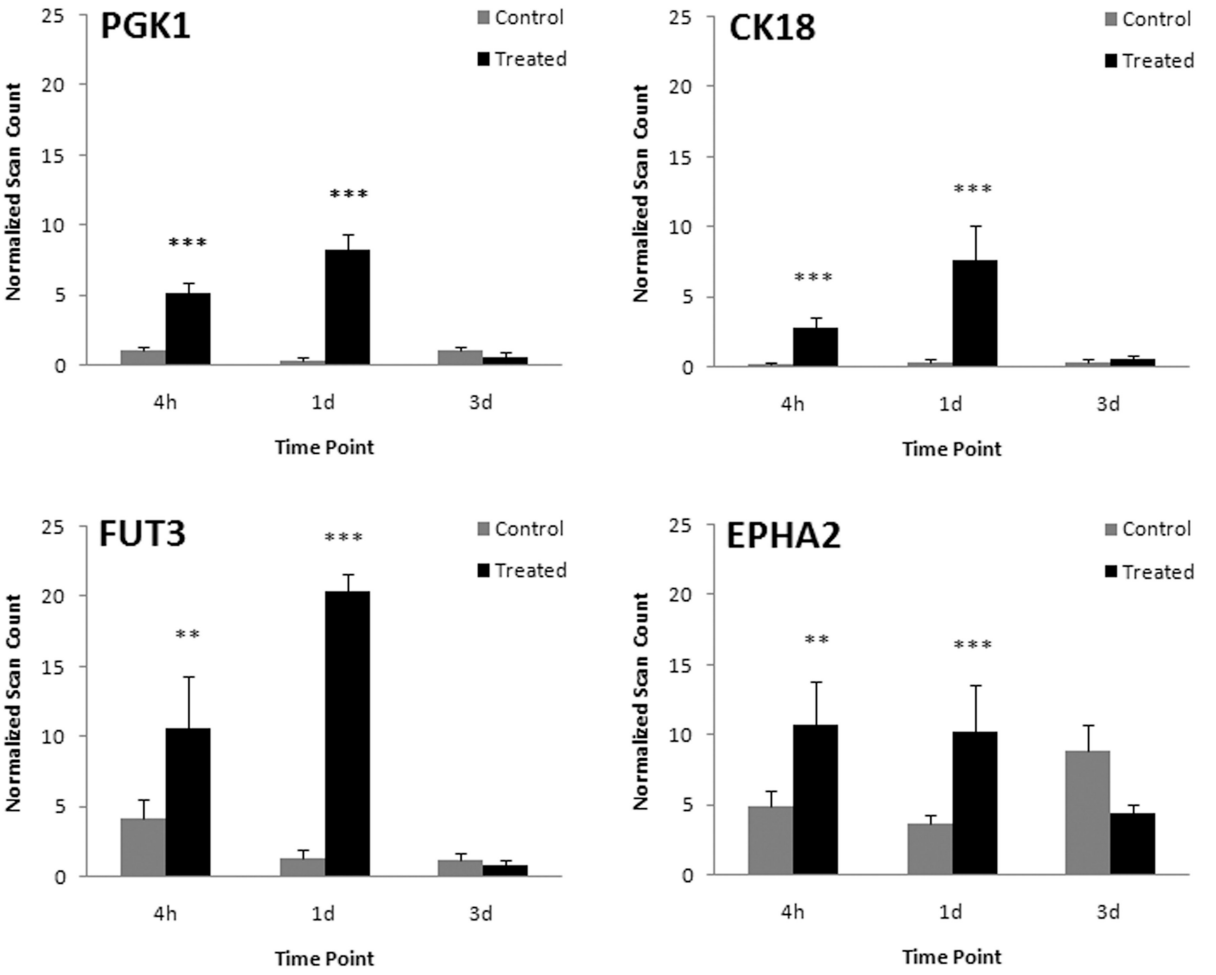
Proteins with significantly higher spectral counts at two time points following laser treatment. Counts are represented as mean number of normalized scans±SEM for control (n=6) and treated samples (n=6) at 4 h (4 h), 1 day (1 day), and 3 days (3 days) following either mock or laser treatment. *p≤0.05, **p≤0.01, ***p≤0.001, for significant differences in treated samples compared with corresponding controls at each time point.

Sera from the 4 h and 1 day time points had four proteins in common that were detected at significantly higher levels in treated samples compared to mock samples (Figure 2). These included phosphoglycerate kinase 1 (PGK1), keratin 18 (CK18), Lewis alpha-3-fucosyltransferase (FUT3), and ephrin receptor A2 (EPHA2). Additionally, these proteins showed high reproducibility for detection, having been identified in all six treated samples at both time points.

Six proteins were detected in either the 4 h or 1 day sera of treated animals only. In all cases, these proteins were also detected in both control and treated samples at the other time points, but the differences were not significant.

### Gene ontology

The 19 proteins identified by spectral counting as potential biomarkers were classified according to gene ontology (GO) terms for both cellular components and biologic processes (Table 2). Eight of the proteins were classified as being integral to the cell membrane, six of which have roles in signal transduction and two of which are involved in the immune response. Two proteins were classified as specific to organelle membranes (Golgi and endoplasmic reticulum). Five proteins were identified that localize in the cytoplasm, while three others localize in the extracellular region. Four of the proteins have association with the post-translational modification function and two play a role in the inflammatory response. One cytoplasmic protein, keratin 18, is an intermediate filament of the cytoskeleton and was classified in terms of its biologic process as having an association with the regulation of apoptosis.

**Table 2:** Gene ontology. Proteins with significantly higher spectral counts in serum following laser treatment compared with mock control, organized by gene ontology (GO) terms for cellular localization and biologic process.

Protein description	Biological process GO term	Cellular component GO term
Alpha-1D adrenoceptor (ADRA1D)	GO:0007165-Signal transduction	GO:0016021-Integral to membrane
Beta-1 adrenergic receptor (ADRB1)	GO:0007165-Signal transduction	GO:0016021-Integral to membrane
Ephrin receptor A2 (EPHA2)	GO:0007165-Signal transduction	GO:0016021-Integral to membrane
Taste receptor type 2 (TAS2R5)	GO:0007165-Signal transduction	GO:0016021-Integral to membrane
Nicotinic receptor alpha 5 subunit (CHRNA5)	GO:0007165-Signal transduction	GO:0016021-Integral to membrane
Alpha-1A adrenoceptor (ADRA1A)	GO:0007165-Signal transduction	GO:0016021-Integral to membrane
Toll-like receptor 8 (TLR8)	GO:0006955-Immune response	GO:0016021-Integral to membrane
MHC class I antigen (HLA-B)	GO:0006955-Immune response	GO:0016021-Integral to membrane
Lewis alpha-3-fucosyltransferase (FUT3)	GO:0043687-Post-translational protein modification	GO:0031090-Organelle membrane
Cytochrome P450, 3A43 (CYP3A43)	GO:0055114-Oxidation reduction	GO:0031090-Organelle membrane
Phosphoglycerate kinase 1 (PGK1)	GO:0043687-Post-translational protein modification	GO:0005737-Cytoplasm
Cereblon (CRBN)	GO:0043687-Post-translational protein modification	GO:0005737-Cytoplasm
Tyrosine kinase-2 (TYK2)	GO:0043687-Post-translational protein modification	GO:0005737-Cytoplasm
Keratin 18 (CK18)	GO:0006915-Apoptosis	GO:0005737-Cytoplasm
28kDa calbindin 1 (CALB1)	GO:0048167-Regulation of synaptic plasticity	GO:0005737-Cytoplasm
Protein phosphatase 1, regulatory subunit 10 (PPP1R10)	GO:0006350-Transcription	GO:0005634-Nucleus
C-C motif chemokine 5 (CCL5)	GO:0006954-Inflammatory response	GO:0005576-Extracellular region
Complement factor 4 (C4)	GO:0006954-Inflammatory response	GO:0005576-Extracellular region
Tissue factor pathway inhibitor (TFPI)	GO:0007596-Blood coagulation	GO:0005576-Extracellular region

## Discussion

In this study, we describe a panel of candidate protein biomarkers that appear to be considerably elevated in response to acute laser-induced injury of the retina. We used a global proteomics approach to analyze serum, along with a stringent data filtering method, to detect and identify the proteins most consistently elevated following laser treatment.

Liquid chromatography/tandem mass spectrometry-based shotgun proteomics is a highly sensitive technique to detect alterations in the proteome of diseased tissue or body fluid. Proteomic analysis of complex mixtures, such as serum, can be challenging in terms of the sensitivity of detection. Therefore, we employed pre-fractionation of serum by isoelectric focusing to increase detectability of potentially low abundant protein candidates. Label-free quantitative proteomics is based on the assumption that increased abundance of a specific protein will lead to increased spectral counts of its tryptic peptides identified by LC/MS/MS. The resultant observable indices include chromatographic ion peak intensity, sequence coverage, peptide number, and spectral count [26]. We used the spectral counting approach for the relative quantification of proteins in serum. Comparison of the total number of MS/MS spectra detected for a given protein (spectral counting) is a reliable and highly reproducible method for relative quantitation. In fact, it has been demonstrated that spectral count is the factor with the strongest correlation (r^2^=0.9997) to protein abundance [27]. We used the G statistic to assess significant differences and then applied a post-hoc Holm-Sidak adjustment of the p value to correct for multiple testing. The G-test was found to be the most appropriate method of statistical analysis based upon our experimental design [24,28]. We applied additional layers of stringency to the data filtering process to minimize false identifications, to identify proteins that were significantly upregulated following laser injury, and to increase our confidence in the final protein biomarker candidate list.

Since the retinal damage was localized to the RPE photoreceptor complex, we expected that photoreceptor- and RPE-specific proteins would be detected in the serum. For this to be the case, one presumes these proteins survive the laser assault intact, are shed from cells, and pass into the circulation system in detectable amounts. Our inability to detect any retina-specific proteins in serum following laser injury might be attributed to several causes. These proteins may have been more vulnerable to degradation either at the site of laser injury or upon entering the systemic circulation. Also, if retina-specific proteins were indeed present in serum, their concentrations may have been far too low for detection using this mass spectrometry approach.

The candidate protein biomarkers identified in this study represent a wide array of proteins that were elevated to significant levels in the serum during the early phases following injury, but that appeared to rapidly return to basal levels. These proteins may have been upregulated locally in the retina at the site of injury and may have entered the systemic circulation through a compromised blood-retina barrier (BRB). Retinal laser injury could lead to induction of pathways for response to ischemia, inflammation, or cell death [8,9]. Events such as these, resulting in protein upregulation, might explain the presence of detectable levels in serum. Gene ontology analysis revealed four proteins with common biologic processes that are known to participate in immune responses. Two of these proteins, C-C motif chemokine 5 (CCL5) and Complement factor 4 (C4), are more specifically classified as inflammatory response proteins. C-C motif chemokine 5 is expressed in the RPE and may have a role in the migration of inflammatory cells across the BRB [29,30]. Increased expression of CCL5 has been associated with autoimmune uveitis [31,32] and has been shown to be a potential serum marker of diabetic retinopathy [33]. The idea that chemokine production at the BRB may increase in response to retinal injury, controlling infiltration of immune cells, and may be detectable at the systemic level, is of particular interest and requires further investigation. We also observed that the temporal profile of the biomarker response may correspond to both the gene expression changes shown to occur following retinal injury [34] and the initial tissue changes after laser injury reported by Tso et al. [5,7,35]. Photoreceptor mummification and RPE necrosis from thermal damage, ischemia resulting from damage to the choriocapillaris, and disruption of the outer BRB, all histologically observed at 1 day post-injury, coincides with our observation of maximum protein biomarker elevation in serum. This type of early response is similar to that described following traumatic brain injury (TBI) or during ischemic events in the brain, where a rapid and transient upregulation of proteins marks the acute-phase reaction [36-38]. The most successful surrogate marker of TBI, protein S-100B, when measured in serum has been shown to be useful in assessing injury severity [39] and as a reliable indicator of blood-brain barrier disruption [40].

Four proteins in our analysis were significantly elevated at both the 4 h and 1 day time points when compared to the serum from controls and the protein levels returned to baseline after 3 days. The proteins included CK18, PGK1, FUT3, and EPHA2. At 4 h following injury, these proteins were detected as being significantly elevated in the serum and then showed a trend toward further elevation after 1 day, suggesting a continuous release of proteins with changes in the biomarker profile for up to 24 h following laser treatment. Although other proteins that showed significant change at only one time point may indeed be of importance, the changes seen with CK18, PGK1, FUT3, and EPHA2 are most apparent and are therefore discussed in detail below.

CK18 is one of the four proteins that showed a transient significant increase in the serum following retinal laser injury. The protein, a component of the cytoskeleton belonging to the intermediate filament family of proteins, is expressed mainly in epithelial cells [41]. In the retina, CK18 expression is restricted to the RPE and is the dominant cytokeratin type in those cells [42-44]. This specificity makes CK18 an effective marker for RPE cell identification [42]. It has been shown that cleavage of CK18 by caspases is an early apoptotic event [45]. Numerous reports suggest that caspase cleaved fragments of CK18, because they can be detected in serum, are a useful indicator of epithelial cell death [46-48]. Our data showed significant detection of CK18 peptides in the serum of laser-treated animals. The peptides detected were tryptic peptides corresponding to a region within the caspase cleaved CK18 fragment (data not shown). We suspect that acute damage of RPE cells induced either a necrotic or apoptotic mode of cell death and that the resulting CK18 fragments are detectable upon release into the serum. Additional studies of the laser dose response and other variables are needed to determine the detection thresholds for such a biomarker in the serum.

PGK1 is an ubiquitous glycolytic enzyme that catalyzes the conversion of 1,3-diphosphoglycerate to 3-phosphoglycerate [49]. In the retina, PGK1 appears to be upregulated in maturing photoreceptors [50] and PGK1 deficiency has been implicated in one case of retinitis pigmentosa [51]. It is likely this enzyme is abundant in metabolically active tissue, such as the retina, and may be the source of the PGK1 seen in the serum after laser injury. PGK1 is upregulated in response to hypoxia [52,53] and during hypoxia-induced apoptosis in cultured retinal neurons [54]. It is therefore feasible that upregulation of PGK1 in the retina might have occurred in the area surrounding the laser injury. It remains unclear whether such an increase in expression would contribute to elevated PGK1 serum levels within 4 h and 1 day after laser exposure, but it is likely released as a direct result of tissue damage in the retina.

EPHA2 belongs to a subfamily of receptor protein-tyrosine kinases. Studies have shown it is expressed mainly in the retinal ganglion cells, but also in amacrine cells and the outer retina during development [55,56]. It plays a role in the development of the retinotectal projection system and is also expressed in the adult retina [55-57]. Following retinal laser injury, altered expression is observed for both ephrin A2 in the superior colliculus and EPHA5 in the retina [58]. Our observation of increased levels of EPHA2 in the serum following retinal laser injury, affecting mainly the outer retina, is somewhat unexpected. It is possible that some injury may have occurred to amacrine cells following the laser injury, which was not clearly evident during our histological assessment of one animal. Also, since EPHA2 is upregulated in the region of growth cones, laser injury may stimulate its production in the outer retina. It is possible that elevated levels of EPHA2 in the inner retina diffuse through a disrupted BRB in response to injury and enter the systemic circulation, allowing detection in the serum. Further studies of the retinal expression of ephrin receptor A2 and additional validation is needed before any definite conclusions can be made regarding this finding.

FUT3 is the enzyme that catalyzes the addition of fucose to precursor polysaccharides in the last step of Lewis antigen biosynthesis [59]. The Lewis histo-blood group system comprises a set of fucosylated glycosphingolipids that are synthesized by exocrine epithelial cells and that circulate in body fluids. Additionally, these glycosphingolipids function in embryogenesis, tissue differentiation, tumor metastasis, inflammation, and cell adhesion [60-62]. Evidence exists for increased FUT3 activity and the resultant increased abundance of Lewis antigen-bearing glycolipids during hepatic inflammation [63], neural differentiation [64], and in epithelial cancer cells [60,65]. It is also known that these fucosylated glycolipids are ligands for E-selectin receptors [66]. Because E-selectin is expressed in retinal vascular endothelial cells and has a role in recruiting inflammatory cells across the BRB [67], it is possible that increased expression and activity of the fucosyltransferases might occur at a retinal injury site. This connection between E-selectin and fucosyltransferases could be relevant to our case of retinal injury if RPE cells do in fact express FUT3. Although the preferential expression of FUT3 in epithelia is known, no sources discuss its activity in the RPE. It would therefore be necessary to assess the presence of FUT3 in RPE and identify an increased expression following retinal injury. The reason for elevation of this enzyme in the sera following retinal injury remains unclear. One could suggest that this response is either related to early-phase inflammatory processes or is a result of FUT3 upregulation followed by release from laser-damaged RPE cells. Further investigation into this response is warranted.

This study is unique in that there are no comparator studies of a similar nature that address acute retinal injury. Most reported studies have used proteomic techniques to investigate alterations in the retinal proteome as a result of diabetes, age-related macular degeneration, or glaucoma. Some studies have assessed the proteomes of intraocular fluids, such as the aqueous humor, and were able to demonstrate significant alterations at the tissue level. However, few studies have been able to demonstrate potential biomarkers of ocular disease in serum and there are none, to our knowledge, relating to acute injury. Great difficulty is typically encountered when attempting to detect potentially low abundance proteins in a complex biologic fluid such as serum. Our ability to identify protein biomarker candidates was perhaps limited by the very nature of using a shotgun proteomics technique. These intrinsic limitations include threshold of detection, under sampling, and sample complexity [68]. Although we employed a strategy of serial fractionation to increase the protein identification rate, the possibility exists that retina-specific proteins may have been present in the serum but were missed due to any of these limitations. We also considered whether the biomarker response we observed was specific to retinal laser injury or if a similar response could occur following laser injury of other structures, such as the trabecular meshwork or iris. Experiments to address this question would be difficult to design. Attempts to replicate laser injury in other tissues with effects that mimic those seen in the retina could be inconsistent, especially if trying to control for laser energy levels and number of spots. Additionally, laser-tissue interactions in the retina and the observed effects are different from those elicited in other ocular tissues. This suggests that the response, although yet to be determined, may be different. Despite these limitations, the technique has proven useful for comparative global protein profiling of serum and we were able to identify biomarker candidates that may be significantly indicative of acute retinal injury. Further investigation focusing on individual candidate proteins is necessary.

## Appendix 1. Comprehensive lists of identified proteins, significantly detected by 2 or more non-redundant peptides, before data filtering.

To access the data, click or select the words “Appendix 1.” This will initiate the download of a compressed (pdf) archive that contains the file.
